# Distribution of trichodorid species in mainland China with description of *Trichodorus hangzhouensis* sp. nov. (Nematoda, Triplonchida)

**DOI:** 10.3897/zookeys.945.50424

**Published:** 2020-07-03

**Authors:** Xuqing Li, Munawar Maria, Ruihang Cai, Eda Marie Barsalote, Vlada Peneva, Jingwu Zheng

**Affiliations:** 1 Laboratory of Plant Nematology, Institute of Biotechnology, College of Agriculture & Biotechnology, Zhejiang University, Hangzhou 310058, Zhejiang, China Zhejiang University Hangzhou China; 2 Hangzhou Academy of Agricultural Sciences, Hangzhou 310024, China Hangzhou Academy of Agricultural Sciences Hangzhou China; 3 Institute of Biodiversity and Ecosystem Research, Bulgarian Academy of Sciences, 2 Gagarin Street, 1113 Sofia, Bulgaria Institute of Biodiversity and Ecosystem Research, Bulgarian Academy of Sciences Sofia Bulgaria; 4 Key Lab of Molecular Biology of Crop Pathogens and Insects, Ministry of Agriculture, Hangzhou 310058, China Ministry of Agriculture Hangzhou China

**Keywords:** 18S rRNA gene, D2-D3 28S rRNA gene, ITS2 region, host association, morphology, new species, phylogeny, taxonomy, Trichodoridae

## Abstract

Seven trichodorid species including a new one (*Trichodorus hangzhouensis***sp. nov.**, *T. nanjingensis*, *T. pakistanensis*, *T. cedarus*, *Paratrichodorus porosus*, *Nanidorus renifer* and *N. minor*) were recovered from the rhizosphere of different hosts in 13 provinces of China. Each of the recovered species was characterized based on morphology and molecular data using rRNA gene sequences. *Trichodorus hangzhouensis***sp. nov.** is characterized by its males having medium-sized onchiostyle (46–49 µm) and three ventromedian cervical papillae (CP) anterior to the secretory-excretory (S-E) pore, CP1 located opposite the anterior part of isthmus, S-E pore opposite the isthmus or anterior end of pharyngeal bulb, spicules slightly ventrally curved, relatively small, 33.2 (32.0–34.5) µm long, wider slightly marked capitulum, lamina partially striated without bristles at striation; and females having rounded triangular sclerotized vaginal pieces with tips directed towards vulva, 1.5–2.0 µm sized, at about 1 µm apart, vulva pore-like in ventral view. Phylogenetic analysis based on D2-D3 28S rRNA gene sequences differentiated the new species among *Trichodorus* species from Europe, Asia and USA which formed a large clade. A review of the distribution of *Trichodorus*, *Nanidorus* and *Paratrichodorus* species revealed that *T. cedarus*, *T. nanjingensis*, *T. pakistanensis* and *P. porosus* are the most widespread species recorded from different provinces of China. This is the first extensive study of trichodorid species occurring in China.

## Introduction

Stubby root nematodes of the family Trichodoridae Thorne, 1935 are polyphagous root ectoparasites and have a global distribution (Decraemer and Robbins 2007). They cause damage to a wide range of crops and natural vegetation by directly feeding on root hairs and epidermal cells. Additionally, some of the species have the ability to transmit plant pathogenic viruses (e.g., Tobacco Rattle Virus (TRV), Pea Early Browning Virus (PBV) and Pepper Rinspot Virus (PRV)) which has stimulated experts to focus on the family Trichodoridae (MacFarlane et al. 2002, Decraemer et al. 2013, 2019). Currently, the family Trichodoridae contains 117 species and six genera (Xu and Zhao 2019, Decraemer et al. 2019). The three didelphic genera (*Trichodorus* Cobb, 1913 (67 spp.), *Paratrichodorus* Siddiqi, 1974 (29 spp.), and *Nanidorus* Siddiqi, 1974 (6 spp.) contain the virus vector species while the rest three monodelphic genera (*Monotrichodorus* Andrássy 1980 (5 spp.), *Allotrichodorus* Rodriguez-M, Sher & Siddiqi, 1978 (8 spp.), and *Ecuadorus* Siddiqi, 2002 (2 spp.) contain few species which are not considered as potential pathogens (Decraemer et al. 2019, Subbotin et al. 2019).

Previously, morphological identification alone rendered difficulties due to the mixed species complexes, phenotypic variation (such as shape of some sclerotized structures, e.g., stylet, male spicules, vagina with its sclerotized pieces), overlapping diagnostic characters and uniformity in general appearance; however, DNA-based strategies have made it possible to overcome the limitations of the morphological approach only and provided useful insights into trichodorid taxonomy (Subbotin et al. 2019). In recent years several nematologists have successfully applied rRNA genes sequence (18S, 28S, and ITS) analyses for studying the phylogenetic relationships of trichodorid species (Duarte et al. 2010, Zhao et al. 2013, Pedram et al. 2015, 2017, Decraemer et al. 2019). The importance of sequence-based studies for species identification and the lack of molecular data of known species from China has led us to compile a detailed report of trichodorids occurring in the country.

The agricultural land of China represents 10% of the total arable land in the world. About 75% of the lands are cultivated areas used for food production such as rice, wheat, potatoes, tea, soybean, various fruits, tea and sugarcane (Guo 2008). Over the past years, there have been preliminary surveys of trichodorid nematodes in China associated with some of the above-mentioned crops, however many of the occurrence records of these nematodes were incomplete or overlooked. Yin and Feng (1981) reported *Trichodorus* species from the southern provinces of Guangdong and Hunan, however, the first official trichodorid survey started with Liu and Cheng (1990). Gradually, several studies on the distribution of trichodorids in China have been published with records from Fujian, Yunnan, Zhejiang and Guangdong provinces reporting the occurrence of six *Trichodorus*, two *Nanidorus* and two *Paratrichodorus* species (Xu and Decraemer 1995, Zheng et al. 2004, Zhao et al. 2005, Chi et al. 2011).

Considering the potential importance of trichodorids in China, an extensive survey of various biotopes was carried out during the recent years. The objectives of the present study are to: i) characterize morphologically and molecularly recovered trichodorid species including one new *Trichodorus* species; ii) evaluate the phylogenetic relationships of the new species with other members of the genera based on their 18S rRNA, D2-D3 expansion domain of 28S rRNA and ITS2 of rRNA gene sequences and iii) summarize the geographic distribution of *Trichodorus*, *Nanidorus* and *Paratrichodorus* species in China, in addition, providing a comprehensive list of the past records and present findings of trichodorid nematodes.

## Materials and methods

### Soil sampling, nematode extraction, and morphological identification

Two-thousand and fifty-two soil samples have been collected from 13 provinces of China. Nematodes were extracted from soil samples using a modified Baermann funnel method and modified Cobb’ sieving and flotation-centrifugation method (Jenkins 1964). For morphological studies, nematodes were killed by hot formalin solution and processed to glycerine according to Seinhorst (1959) as modified by De Grisse (1969). Morphological observation, measurements, and photomicrographs were made using a Leica CTR 5000 compound microscope with differential interference contrast (DIC). Measurements were expressed as mean ±standard deviation (range). Species diagnoses were made following the polytomous key of Decraemer and Baujard (1998). Original descriptions were used for species added to trichodorid genera.

### DNA Extraction, PCR, and sequencing

DNA was extracted from single specimens of all seven recovered species as described by Zheng et al. (2003). Four sets of primers (synthesized by Invitrogen, Shanghai, China) were used in the PCR analyses to amplify the partial 18S, D2-D3 region of 28S and ITS2 region of rRNA gene. The 18S rRNA gene was amplified with the forward primer A (5'-AAA GAT TAA GCC ATG CAT G-3') (Boutsika et al. 2004) and the reverse primer S3 (5'-AGT CAA ATT AAG CCG CAG-3') (Waite et al. 2003). The D2-D3 region of 28S rDNA gene was amplified with the forward primer D2A (5'- ACA AGT ACC GTG AGG GAA AGT TG-3') and the reverse primer D3B (5'-TCG GAA GGA ACC AGC TAC TA-3') (De Ley et al. 1999). The ITS2 region was amplified as two partially overlapping fragments, for the first fragment, the forward primer 18S (5'-TTG ATT ACG TCC CTG CCC TTT-3') (Vrain et al. 1992) and the reverse primer ITSB (5'-GCT GCG TTC TTC ATC GAT-3') (Boutsika et al. 2004) were used, and for the second fragment, the forward primer ITSA (5'-ATC GAT GAA GAA CGC AGC-3') (Boutsika et al. 2004) and the reverse primer 26S (5'-TTT CAC TCG CCG TTA CTA AGG-3') (Vrain et al. 1992) were used. PCR conditions were as described by Ye et al. (2007). PCR products were separated on 1.5% agarose gels and visualized by staining with ethidium bromide. PCR products of sufficiently high quality were purified for cloning and sequencing by Invitrogen, Shanghai, China.

### Phylogenetic analyses

The partial sequences of 18S, D2–D3 fragment of 28S and ITS2 of rRNA gene of *Trichodorus hangzhouensis* sp. nov. were compared with those of other species of fam. Trichodoridae available in GenBank using the BLAST homology search program. The sequence data sets used in this study were selected based on previously published studies (Zeng et al. 2014, Decraemer et al. 2019, Subbotin et al. 2019) and were used in phylogenetic analyses. Three separate 18S, 28S and ITS2 datasets were prepared. Multiple sequence alignment of each dataset was made using the Q-INS-i algorithm of MAFFT V.7.205 (Katoh and Standley 2013). The sequence alignments were edited by BioEdit (Hall 1999). The best fitted model of DNA evolution was obtained using jModelTest V.2.1.7 (Darriba et al. 2012) with the Akaike information criterion (AIC). General time-reversible model with invariable sites and a gamma-shaped distribution (GTR + I + G) was used for the 28S, 18S and ITS2 rRNA genes to reconstruct the phylogenies. Bayesian analysis was used to infer a phylogenetic tree by MrBayes 3.1.2 (Ronquist and Huelsenbeck 2003).

Model parameters were unlinked and the overall rate was allowed to vary across partitions. The number of generations for the total analysis was set to 10 million, with the chain sampled every 1000 generations and the burn-in value was 25%. The Markov chain Monte Carlo method within a Bayesian framework was used to estimate the posterior probabilities of the phylogenetic trees using the 50% majority rule (Larget and Simon 1999). Posterior probabilities (PP) were given on appropriate clades. The consensus trees were visualized using FigTree V1.4.3 (Stöver and Müller 2010).

## Taxonomy

### Trichodoridae Thorne, 1935

#### Trichodorus hangzhouensis sp. nov.

Taxon classificationAnimaliaTriplonchidaTrichodoridae

266EF146-DD2B-5F0F-AE92-4101D1F488A0

http://zoobank.org/F0E3BA04-6CB9-4333-9477-1E2DCB844DB7

##### Description

**Figure 1.**
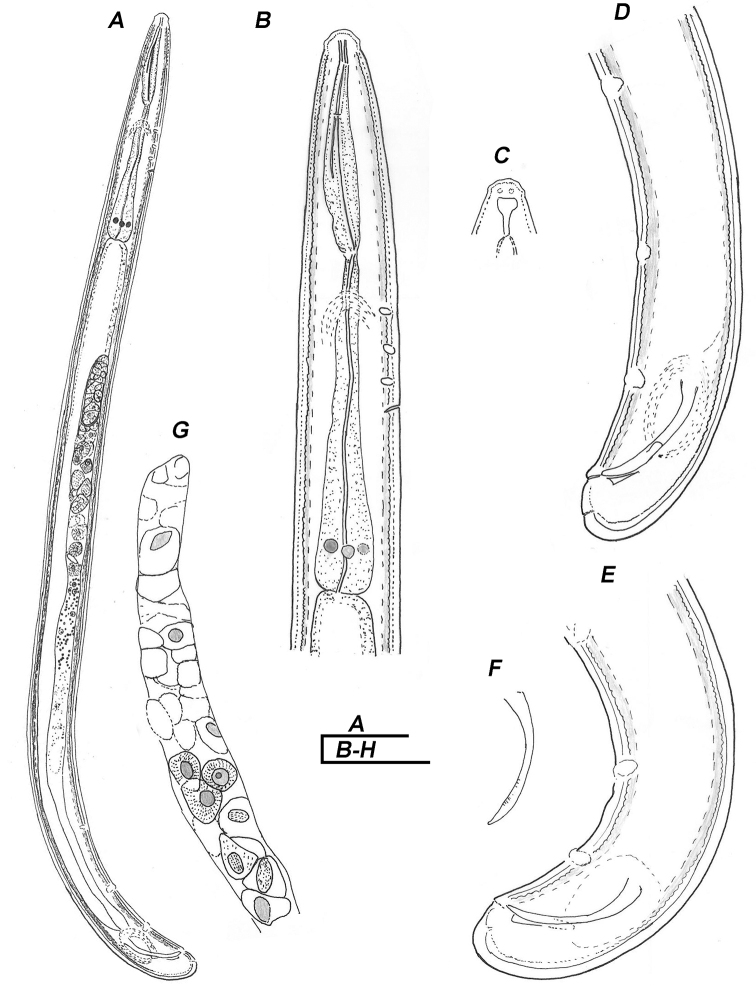
Line drawings of *Trichodorus hangzhouensis* sp. nov., paratypes, Male **A** entire body **B** pharyngeal region **C** surface view **D, E** posterior end **F** spicule **G** germinal zone of testis. Scale bars: 50 μm.

**Figure 2.**
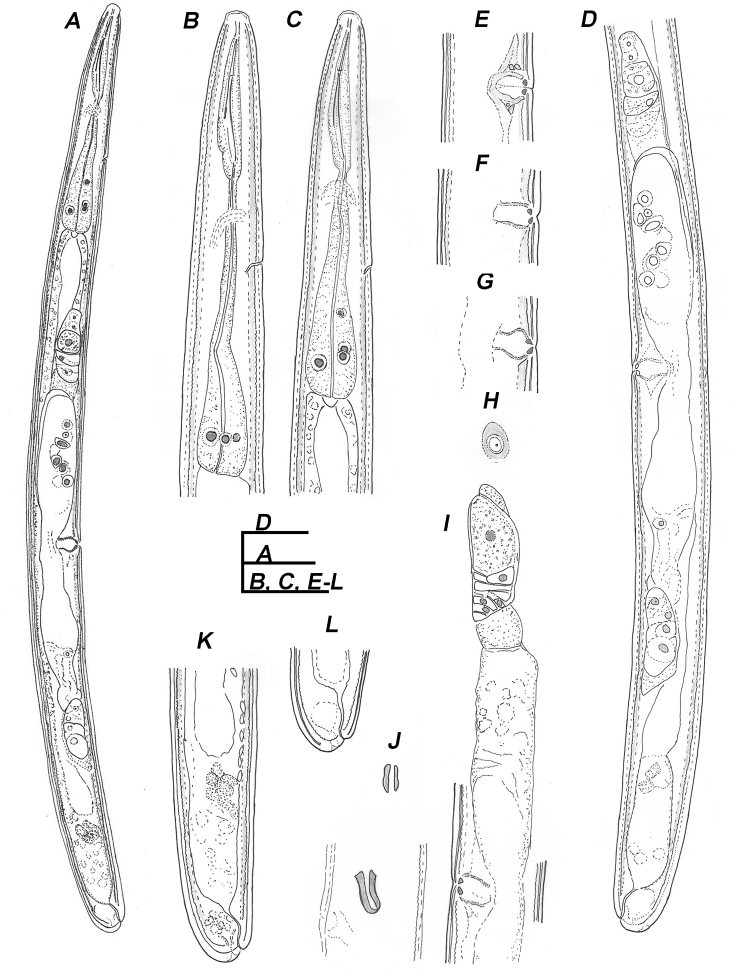
Line drawings of *Trichodorus hangzhouensis* sp. nov., paratypes, Female **A** entire body **B, C** pharyngeal region **D** genital branches **E–G** vulval region, lateral view **H** vulva, ventral view **I** anterior genital branch **J** copulatory plugs **K, L** posterior ends. Scale bars: 50 μm.

**Figure 3.**
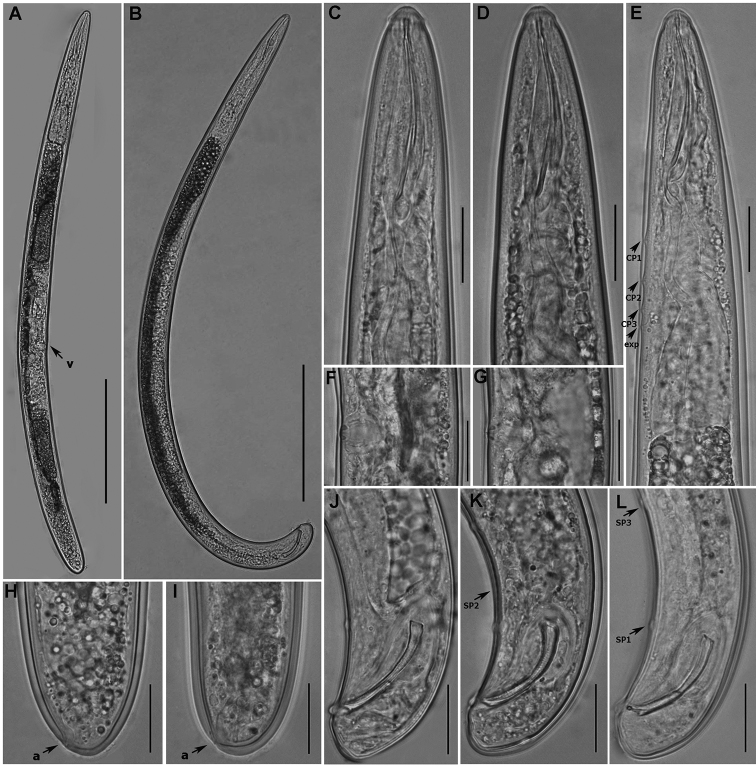
Light photomicrographs of *Trichodorus hangzhouensis* sp. nov., paratypes **A** female entire body, arrow points vulva **B** male entire body **C, D** anterior region of female **E** pharyngeal region of male, arrows pointing position of cervical papillae (CP) and excretory pore (exp) **F, G** vulval region **H, I** female tail, arrows pointing position of anus (a) **J–L** male tail, arrows pointing position of male tail supplements (SP). Scale bars: 100 μm (**A, B**) 20 μm (**C–L**).

(Figs 1–3, For measurements see Table 1). **Male.** Body cylindrical with posterior end slightly curved ventrally. Cuticle slightly swollen upon fixation, 2.0–2.5 µm thick at mid-body. Lip region dome-shaped with double papillae (composed of outer labial and cephalic papillae). Amphidial aperture post-labial, slit-like, amphidial fovea cup-shaped. Stoma narrow, refractive strengthening rods 4–5 µm long. Nerve ring surrounding the anterior part of isthmus. Slender mid part of pharynx gradually widening to form a pharyngeal bulb. Five pharyngeal gland nuclei visible, the first ventrosublateral pair obscure. Pharyngeal bulb offset from intestine. Cardia conoid, difficult to observe. Three ventromedian CP present anterior to the secretory-excretory pore (S-E), the latter opposite isthmus or anterior end of pharyngeal bulb. CPl situated opposite the end of pharyngostom to mid-isthmus, distance of CPl-CP2, CP2-CP3 and CP3-SE becomes gradually shorter. Lateral cervical pores not clearly seen. Reproductive system typical of the genus, i.e., with a single anterior outstretched testis, short germinal zone, seminal vesicle packed with large round sperm cells with fibrillar structure and a sausage-shaped central nucleus. Spicules paired, relatively short 33.2 (32.0–34.5) µm, in holotype 34.5 µm, slightly ventrally curved. Capitulum widened, slightly marked, lamina partially striated, tapers gradually to the distal end, no bristles at striation. Gubernaculum having a keel-like thickening and proximal end visible between spicules (Fig. 1E). Three ventromedian precloacal supplements (SP) present. The posterior-most one (SP1) at the level of spicule capitulum, the SP2 slightly less than, or equal to one spicule length anterior to the SP1. The anterior most (SP3), 1.0–1.5 times spicule length apart from SP2. Cloacal lip rounded; slightly protruded, post-cloacal papillae not prominent. Tail short, conoid, less than one cloacal diameter long with one pair of subterminal subventral pores.

**Female.** Body straight or slightly curved upon heat relaxation. Anterior region similar to that of male except for secondary male characteristics. S-E pore located opposite isthmus or anterior part of pharyngeal bulb. Reproductive system didelphic amphidelphic with reflexed ovaries. Two finely granular oviduct cells at the tip of reflexed ovary, sperm round in shape distributed in the distal part of the uteri. Vagina well developed, *pars proximalis vaginae* barrel shaped in lateral optical view extending less than half corresponding body diameter. Sclerotized vaginal pieces (= *pars refringes vaginae*) rounded triangular with tips directed towards vulva, pieces 1.5–2.0 µm sized, at about 1.0 µm distance from each other, vulva pore-like in ventral view. Copulatory plug observed in uterus of two specimens. One pair of sublateral body pores almost opposite the vulva. Tail terminus conoid to rounded, anus subterminal, caudal pores subventral, immediately posterior to anus.

**Table 1.** Measurements of *Trichodorus hangzhouensis* sp. nov. males and females. All measurements are in µm and in the form: mean ± SD (range).

Characters	Holotype	Paratypes	
	Male	Males	Females
n		8	11
L	708	689.9 ± 30.4 (628–727)	686.2 ± 29.8(646–744)
Body diameter	33	33.8 ± 2.2 (29–36)	40.4 ± 3.3 (36.0–47.0)
Pharynx	145	140.8 ± 8.3 (125–146)	143.3 ± 15.0 (117–171)
Onchiostyle	48	48.1 ± 1.2 (46–49)	49.3 ± 1.8 (48–52)
Onchium	27	26.9 ± 2.3 (24–28)	29.4 ± 1.9 (27–31)
Onchiophore	22	21.6 ± 0.7 (21–23)	22.1 ± 0.9 (2–23)
Pharyngostom	53	50.5 ± 2.8 (47–54)	54.6 ± 2.1 (52–56)
Ant. end to S-E pore	99	100.0 ± 4.0 (96.5–108)	96.8 ± 8.2 (87–114)
Ant. genital branch	–	–	174.2 ± 6.8 (163–185)
Post. genital branch	–	–	170.1 ± 15.5 (137–193)
a	21.3	20.5 ± 1.1 (19.0–21.6)	17.1 ± 1.5 (14.5–19.4)
b	4.9	4.9 ± 0.4 (4.3–5.6)	4.8 ± 0.6 (3.8–6.0)
V/T	64.5	65.8 ± 1.4 (63.9–68.4)	57.1 ± 2.0 (53.0–60.0)
Length of vagina	–	–	15.6 ± 1.4 (14–19)
CP1-CP2	12	10.9 ± 1.9 (8–12.5)	–
CP2-CP3	9	7.9 ± 1.7 (5–9)	–
CP3 to S-E pore	8	6.0 ± 1.5 (4–8)	–
Spicules	34.5	33.2 ± 1.0 (32–34.5)	–
Gubernaculum	15	14.7 ± 1.5 (11.5–17)	–
Cloaca to SP1	23	25.7 ± 3.0 (22.5–32)	–
SP1-SP2	24	31.3 ± 4.4 (24–37)	–
SP2-SP3	37.0	38.0 ± 4.0 (33–45)	–

##### Type host and locality.

The new species was detected in association with three plants, i.e., *Eriobotrya japonica* (Thunb.) Lindl., *Morus alba* L. and *Toona sinensis* (A. Jussieu) M. Roemer from the botanical garden of Huajiachi Campus, Zhejiang University, Hangzhou, Zhejiang Province, P. R. China. The specimens from *E. japonica* were regarded as a type population. The geographical position of the sampling site is: 120°19'06"E, 30°25'67"N.

##### Type material.

***Holotype*** male, 8 male and 11 female ***paratypes*** (slide nos. ZJU-29-01-ZJU-29-19) deposited in the Nematode Collection of Zhejiang University, Hangzhou, China, and 2 male and 13 female paratypes deposited in Institute of Biodiversity and Ecosystem Research, Bulgarian Academy of Sciences, Bulgaria (slide nos. PNT 102-104).

##### Molecular profiles and phylogenetic analysis.

The new species was molecularly characterized and newly obtained sequences were deposited in the GenBank with the accession numbers HM106498, MF979178 for 18S, MF979185–MF979186, HM106497 for 28S and MF979181, HM106496, MF979182 for ITS2 of rRNA gene. The available sequences of trichodorid taxa (accession numbers of 18S, D2–D3 region of 28S and ITS2 rRNA gene sequences in Suppl. material 1: Table S1) were selected to reconstruct the phylogenetic trees.

The 18S rRNA gene tree (Fig. 7) revealed that *T. hangzhouensis* sp. nov. occupied a basal placement in an unsupported clade including *T. primitivus* (de Man, 1880) Micoletzky, 1922 (KY119675-76, AF036609), *T. similis* Seinhorst, 1963 (AJ439584-85, AJ439522) and *T. obtusus* Cobb, 1913 (KT282335). The pairwise sequence identity of new species with the aforementioned species is 97–99% with 16–24 nucleotide differences.

In the 28S phylogenetic tree (Fig. 8), *T. hangzhouensis* sp. nov. formed a clade with four species distributed in Spain: *T. giennensis* Decraemer, Roca, Castillo, Peñ-Santiago & Gomez-Barcina, 1993 (JQ716452), *T. illiplaensis* Decraemer, Palomares-Rius, Cantalapiedra-Navarrete, Landa, Duarte, Almeida, Vovlas & Castillo, 2013 (JQ716462), *T. onubensis* Decraemer, Palomares-Rius, Cantalapiedra-Navarrete, Landa, Duarte, Almeida, Vovlas & Castillo, 2013 (JQ716454-55) and *T. paragiennensis* Decraemer, Palomares-Rius, Cantalapiedra-Navarrete, Landa, Duarte, Almeida, Vovlas & Castillo, 2013 (JQ716461); three species occurring in Iran – *T. orientalis* De Waele & Hashim, 1983 (KY115140); *T. persicus* De Waele & Sturhan, 1987 (KX348138); *T. zanjanensis* Asghari, Eskandari, Tanha Maafi & Decraemer, 2018 (KY115138); one species from Israel *T. minzi* De Waele & Cohn, 1992 (KP259801) and an undescribed *Trichodorus* species (KM212949) from the USA. The pairwise sequence identity of new species with the aforementioned species is 88–91% with 73–86 nucleotide differences.

In ITS2 tree (Fig. 9), *T. hangzhouensis* sp. nov. shared the same clade with *T. pakistanensis* Siddiqi, 1962 (GU645896, GU645897, GU645899, JN123384) and the clade of these two species was in sister relation to a clade including *T. gilanensis* Maafi & Decraemer, 2002 (KY115164–KY115165), *T. nanjingensis* Liu & Cheng, 1990 (GU645800, GU645804, GU645893, GU645895), *T. viruliferus* Hooper, 1963 (JN123391) and *T. sparsus* Szczygiel, 1968 (JN123388). The pairwise sequence identity of the new species with the aforementioned species is 94–98% with 4–9 nucleotide differences.

The other known *Trichodorus*, *Nanidorus* and *Paratrichodorus* species sequenced during this study clustered with their respective species available through GenBank database, thus supporting their identity.

##### Diagnosis and relationships.

The new species is characterized by the male having a relatively short onchiostyle (46–49 µm) and 3 ventromedian cervical papillae anterior to the S-E pore, CP1 located opposite isthmus, distance of CPl-CP2, CP2-CP3 and CP3-S-E becoming gradually shorter, S-E pore located opposite isthmus or anterior end of pharyngeal bulb, pharynx offset, spicules relatively short, slightly curved, 33.2 (32.0–34.5) µm long, with wider slightly marked capitulum, lamina partially striated and tapering gradually to the distal end, bristles at striation absent, three ventromedian precloacal supplements; female with barrel shaped vagina, vaginal scletorized pieces medium-sized (1.5–2.0 µm), rounded triangular with tips directed towards vulva, slightly separated from each other (c. 1.0 µm) , vulva pore-like in ventral view.

The species-specific codes sensu Decraemer and Baujard (1998) for this new species are as follows, for the male: F3-D3-P2-A1(2)-B2-C1-E0-G1-H2-I1-J2-K3-L2-M4-N1-O5; for female: D1-C1-L2- K2-A1(2)-B2-E2 -F1 G1-H3-I2(3)-J1-M1-N1-O1-P1-Q1-R2-S5. Based on male prime diagnostic characters for fam. Trichodoridae (F = number of ventromedian precloacal supplements, D = number of ventromedian cervical papillae, P = body habitus) and female (D = type of genital system, C = vulva position, K = size of vaginal sclerotized pieces, L = position of vaginal sclerotized pieces) the new species male belongs to group 12 while the female falls in the category group 1 of subgroup 1–7 as described by Decraemer and Baujard (1998). Among several *Trichodorus* species of group 12 (for male) and group 1, subgroup 1–7 (for female) the new species comes close to *T. cedarus* Yokoo, 1964, *T. guangzhouensis* Xie, Feng & Zhao, 2000, *T. reduncus* Siddiqi & Sharma, 1995, *T. tricaulatus* Shishida, 1979 and *T. yokooi* Eroshenko & Teplyakov, 1975. It can be differentiated as follows:

*T. cedarus* – by having a different position of SP1 (at the level of spicules capitulum vs posterior), shorter spicules length (32.0–34.5 vs 36–53 µm), shape of vulva (pore-like vs slit-like) and shape of vagina (barrel vs pear);

*T. guangzhouensis* – by having a longer onchiostyle in females (48–52 vs 36.4–41.6 μm), striations on spicule (present vs absent), different spicule shape (without constriction vs with constriction) and shape of vulva in ventral view (pore-like vs a longitudinal slit);

*T. reduncus* – by having a longer onchiostyle in males (46–49 vs 36–40 µm) and female (48–52 vs 37–40 µm), striations on spicules (present vs absent), different proximal part of gubernaculum (not hooked vs hooked) position of vaginal pieces (close vs widely separated) and shape of vulva (pore-like vs a small transverse slit);

*T. tricaulatus* – by having different position of SP1 (at the level of spicule capitulum vs outside it), spicule bristles (absent vs present), longer onchiostyle in both males (49.3 (46–49) vs 42.6 (39–51) µm) and females (49.3 (48–52) vs 42.2 (39–44) µm), type of pharyngo-intestinal junction (pharyngeal bulb offset vs pharynx overlapping intestine ventrally) and shorter spicules (33.2 (32.0–34.5) vs 39 (36–53) µm);

*T. yokooi* – by having striation on spicule (vs absent), shorter onchiostyle both in males (46–49 vs 57–82 µm) and females (48–52 vs 62–77 µm) and spicule (32.0–34.5 vs 38–46 µm).

##### Etymology.

The species name is derived from the name of the city where the new species was recovered.

### Distribution of trichodorid species in mainland China

**Figure 4.**
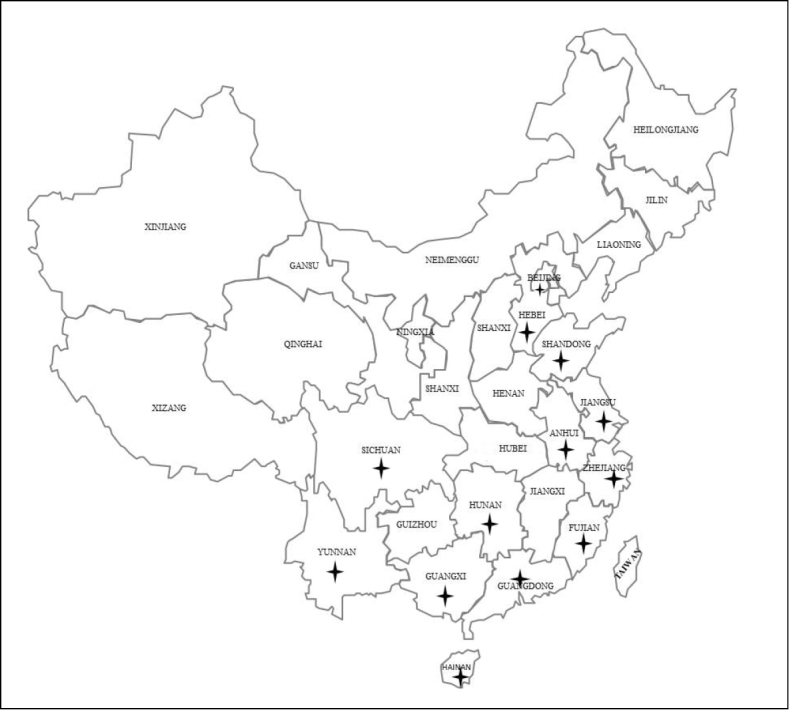
Distribution map of trichodorid species in China (stars indicate the occurrence of trichodorid taxa).

**Figure 5.**
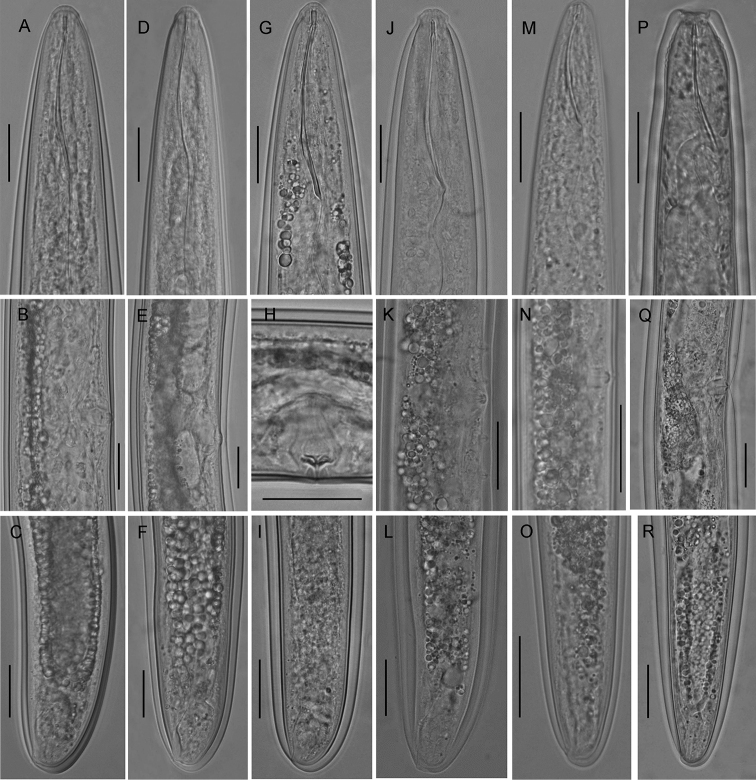
Photomicrographs of females of *Trichodorus*, *Paratrichodorus* and *Nanidorus* species **A–C***T. nanjingensis* Liu & Cheng, 1990 **D–F***T. pakistanensis* Siddiqi, 1962 **G–I***T. cedarus* Yokoo, 1964 **J–L***P. porosus***M–O***N. renifer* Siddiqi, 1974 **P–R***N. minor* (Colbran, 1956). Scale bars: 20 μm (**A–R**).

**Figure 6.**
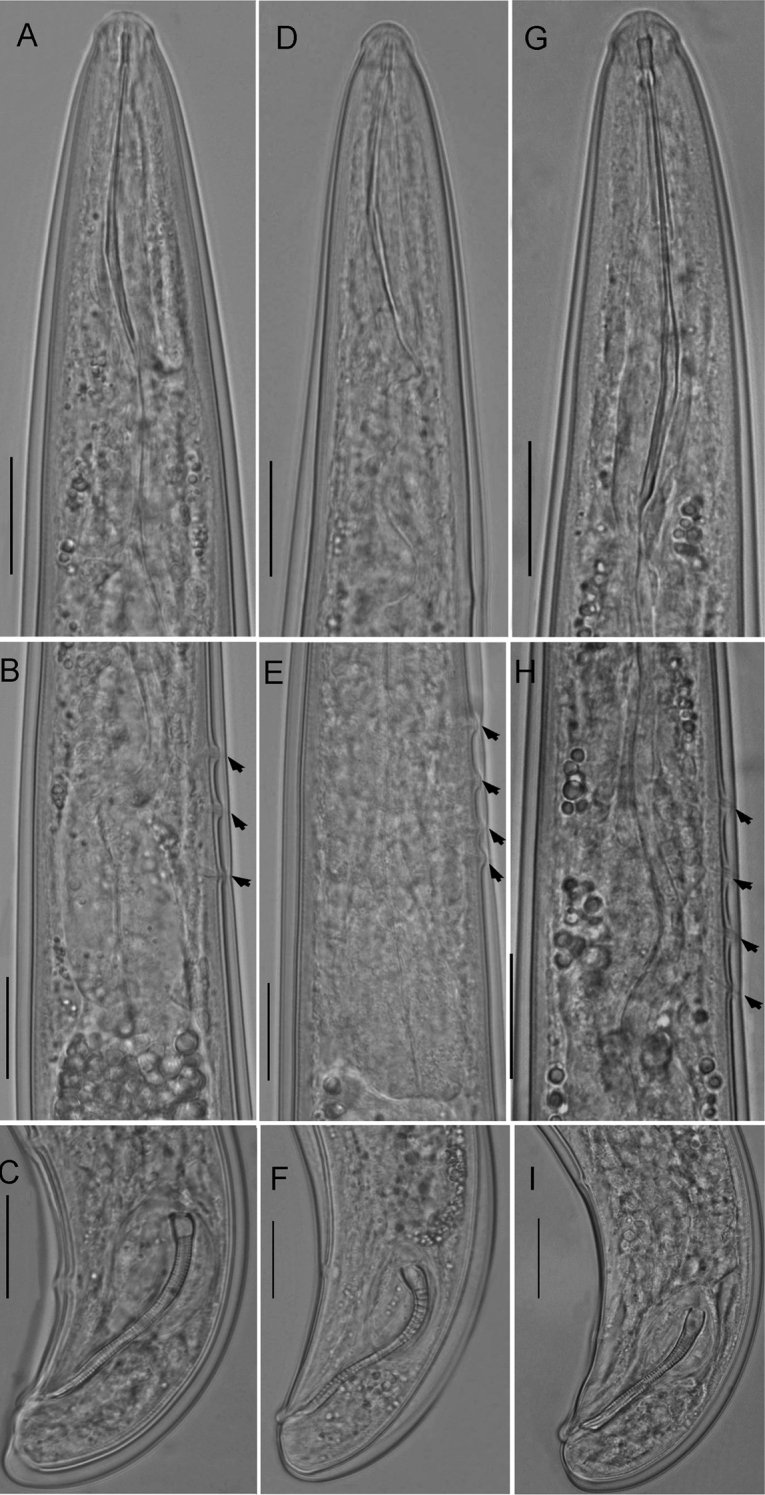
Photomicrographs of *Trichodorus* species, Males **A–C***T. nanjingensis* Liu & Cheng, 1990 **D–F***T. pakistanensis* Siddiqi, 1962 **G–I***T. cedarus*. Scale bars: 20 μm (**A–I**).

The geographical distribution of trichodorids recovered in 13 different provinces of China including Beijing, Shandong, Shanxi, Henan, Jiangsu, Anhui, Hunan, Chongqing, Zhejiang, Fujian, Yunnan, Hainan and Guangdong (based on 2054 examined soil samples) is mapped in Fig. 4. Eighty-five trichodorid populations were recovered in this study while ninety-three populations are listed in previous records from China (Table 2). In this study, three known species of *Trichodorus* (*T. nanjingensis*, *T. pakistanensis*, *T. cedarus*) with one new species, one *Paratrichodoru*s species (*P. porosus* (Allen, 1957) Siddiqi 1974), and two *Nanidorus* species (*N. renifer* Siddiqi, 1974; and *N. minor* (Colbran, 1956) Siddiqi, 1974) have been identified (Figs 5, 6; Tables 3, 4), while *T. paracedarus*, Yokoo, 1964; *T. rinae* Yokoo, 1964; *T. guangzhouensis* and *P. pachydermus* Siddiqi, 1974 were reported in the past (Xu and Decraemer 1995, Xie et al. 2000, Zhao et al. 2005, Yan et al. 2005). In our study 14, 16, 5, 30, 9 and 8 soil samples contain respectively *T. nanjingensis*, *T. pakistanensis*, *T. cedarus*, *P. porosus*, *N. renifer* and *N. minor* accounts for 16.4%, 18.8%, 5.9%, 35.3%, 10.6% and 9.4% of the total trichodorid populations detected. Combined records of past and present reports revealed that the most frequently found *Trichodorus* species are *T. nanjingensis*, *T. pakistanensis* and *T. cedarus*, while *P. porosus* showed remarkably high frequency of occurrence in China. In general, trichodorids have been recovered from many localities in China but some species are geographically concentrated in some areas, e.g., *T. nanjingensis* is recorded in high percentage in the northern area (Beijing), *T. pakistanensis* in the southeastern region (Fujian), *T. cedarus* in eastern regions (e.g., Zhejiang), and *P. porosus* is very common in Yunnan and Zhejiang provinces. Other species were found in a relatively few numbers.

Two of the recorded species are known to transmit tobra viruses (*N. minor* and *P. pachydermus*) (MacFarlane et al. 2002).

**Table 2.** The occurrence of *Trichodorus*, *Paratrichodorus* and *Nanidorus* in China.

Provinces (bold) /Localities	Species	Host	References
**Jiangsu**			
Nanjing	*T. nanjingensis*	*Prunus persica*	Liu and Cheng 1990
Suzhou	*T. nanjingensis*	*Prunus salicina*	Decraemer and Cheng 1994
Linbao	*T. nanjingensis*	*Malus domestica*	Decraemer and Cheng 1994
Ganyu	*T. cedarus*	*Malus pumila*	Xu and Decraemer 1995
Wuxi	*T. cedarus*	*Prunus persica*	Xu and Decraemer 1995
Nanjing	*T. paracedarus*	*Lycopersicon esculentum*	Xu and Decraemer 1995
Lianyungang	*T. paracedarus*	*Prunus yedoensis*	Xu and Decraemer 1995
Nantong	*P. porosus*	*Marus alba*	Liu 1994
**Zhejiang**			
Hangzhou	*T. nanjingensis*	*Bambusa glaucescens*	Zheng et al. 2004
Hangzhou	*T. pakistanensis*	*Metasequoia glyptostroboides*	Zheng et al. 2004
Changxing	*T. cedarus*	*Pyrus pyrifolia*	Xu and Decraemer 1995
Huzhou	*P. porosus*	*Bambusa vulgates*	Xie et al. 2007
Fuyang	*P. porosus*	*Camellia japonica*	Zheng et al. 2004
Ningbo	*T. cedarus*	*Hedera helix*	This study
Ningbo	*P. porosus*	*Ilex chinensis*	This study
Ningbo	*N. renifer*	*Myrica rubra*	This study
Ningbo	*N. renifer*	*Myrica rubra*	This study
Ningbo	*N. renifer*	*Myrica rubra*	This study
Hangzhou	*T. nanjingensis*	*Morus alba*	This study
Hangzhou	***T. hangzhouensis* sp. nov.**	*Albizia julibrissin*	This study
Hangzhou	*T. cedarus*	*Magnolia denudata*	This study
Hangzhou	*T. cedarus*	*Osmanthus fragrana*	This study
Hangzhou	*P. porosus*	*Eriobotrya japonica*	This study
Hangzhou	***T. hangzhouensis* sp. nov.** + *P. porosus*	*Firmiana simplex*	This study
Hangzhou	***T. hangzhouensis* sp. nov.**	*Toona sinensis*	This study
Hangzhou	***T. hangzhouensis* sp. nov.**	*Eriobotrya japonica*	This study
Hangzhou	*T. nanjingensis*	*Pyrus* sp.	This study
Hangzhou	*T. nanjingensis*	*Cinnamomum camphora*	This study
Hangzhou	*T. cedarus*	*Cryptomeria fortune*	This study
Hangzhou	*T. nanjingensis*+*P. porosus*	*Albizia julibrissin*	This study
Hangzhou	*T. nanjingensis*	*Rosa rugosa*	This study
Hangzhou	*T. pakistanensis*	*Magnolia grandiflora*	This study
Hangzhou	*T. cedarus*	*Pseudotsuga sinensis*	This study
Hangzhou	*N. renifer*	*Azalea* sp.	This study
Hangzhou	*P. porosus*	*Gmelina hainanensis*	This study
Fuyang	*P. porosus*	*Camellia japonica*	This study
Linan	*P. porosus*	*Prunus pseudocerasus*	This study
Taizhou	*P. porosus*	*Quercus acutissima*	This study
Yuyao	*N. renifer*	*Myrica rubra*	This study
Yuyao	*N. renifer*	*Myrica rubra*	This study
Lishui	*P. porosus*	*Citrus reticulata*	This study
Jinhua	*N. renifer*	*Rosa chinensis*	This study
Fenghua	*P. porosus*+ *N. renifer*	*Acer truncatum*	This study
Fenghua	*P. porosus*+ *N. renifer*	*Acer palmatum*	This study
**Hainan**			
Anding	*P. pachydermus*	*Saccharum officinarum*	Ding et al. 2015
Danzhou	*P. pachydermus*	*Saccharum officinarum*	Ding et al. 2015
Unknown	*T. pakistanensis*	*Saccharum officinarum*	This study
Danzhou	*N. minor*	*Solanum melongena*	This study
Danzhou	*N. minor*	*Melia azedarach*	This study
Danzhou	*N. minor*	*Lactuca sativa*	This study
**Chongqing**			
Beipei	*T. pakistanensis*	*Trachycarpus fortunei*	This study
**Yunnan**			
Kunming	*T. nanjingensis*	*Pyrus* sp.	Zhao et al. 2005
Kunming	*T. rinae*	*Pyrus* sp.	Zhao et al. 2005
Kunming	*T. cedarus*	*Pyrus* sp.	Zhao et al. 2005
Kunming	*P. porosus*	*Pyrus* sp.	Zhao et al. 2005
Hekou	*T. cedarus*	*Musa* sp.	Du et al. 2010
Kunming	*N. minor*	*Hydrangea macrophylla*	Lin et al. 2009
Chengong	*N. minor*	*Prunus persica*	This study
Dabanqiao	*N. minor*	*Pyrus* sp.	This study
Dabanqiao	*N. minor*	*Pyrus* sp.	This study
Luliang	*P. porosus*	*Pyrus* sp.	This study
Luliang	*P. porosus*	*Pyrus* sp.	This study
Majie	*P. porosus*	*Pyrus* sp.	This study
Majie	*P. porosus*	*Pyrus* sp.	This study
Majie	*P. porosus*	*Solanum tuberosum*	This study
Kunming	*P. porosus*	*Sapindus delavayi*	This study
Kunming	*P. porosus*	*Corylus chinensis*	This study
Kunming	*P. porosus*	*Diospyros kaki*	This study
Kunming	*P. porosus*	*Sophora japonica*	This study
Kunming	*P. porosus*	*Quercus variabilis*	This study
Kunming	*P. porosus*	*Abies holophylla*	This study
Kunming	*T. pakistanensis*	*Acer truncatum*	This study
Kunming	*N. minor*	* Juglansregia *	This study
Kunming	*P. porosus*	*Prunus persica*	This study
Xundian	*P. porosus*	*Pinus massoniana*	This study
**Guangdong**			
Shenzhen	*P. porosus*	*Litchi chinensis*	Wang et al. 1996
Unknown	*T. cedarus*	*Salix babylonica*	Chi et al. 2011
Unknown	*P. porosus*	*Magnoliaceae glanca*	Chi et al. 2011
Unknown	*N. renifer*	*Magnoliaceae glanca*	Chi et al. 2011
Guangzhou	*T. guangzhouensis*	*Lactuca sativa*	Xie et al. 2000
Gaozhou	*P. pachydermus*	*Musa paradisiaca*	Yan et al. 2005
Guangzhou	*P. porosus*	*Osmanthus fragrana*	This study
**Beijing**			
Nankou farm	*T. nanjingensis*	*Malus domestica*	Wang et al. 1996
Nankou farm	*T. nanjingensis*	*Mains baccata*	Hao et al. 1998
Unknown	*T. nanjingensis*	*Malus domestica*	Zheng et al. 2004
Nankou farm	*T. pakistanensis*	*Malus domestica*	Wang 1993
Nankou farm	*T. pakistanensis*	*Malus domestica*	Wang et al. 1994
Unknown	*P. porosus*	*Vitis* sp.	Liu 1994
Shisanling	*T. nanjingensis*	*Prunus persica*	This study
Shisanling	*T. nanjingensis*	*Juglans regia*	This study
Shisanling	*T. nanjingensis*	*Malus pumila*	This study
Fenghuangling	*T. nanjingensis*	*Pyrus* sp.	This study
Zhiwuyuan	*T. nanjingensis*	*Malus micromalus*	This study
Zhiwuyuan	*T. nanjingensis*	*Prunus persica*	This study
Zhiwuyuan	*T. nanjingensis*	*Cotoneaster multiflorus*	This study
Unknown	*T. nanjingensis*	*Prunus blireana*	This study
Xiangshan	*T. nanjingensis*	*Prunus armeniaca*	This study
**Hebei**			
Zhuolu	*P. porosus*	*Vitis vinifera*	Wang et al. 1996
Xingtang	*P. porosus*	*Vitis vinifera*	Wang et al. 1996
**Shandong**			
Linyi	*P. porosus*	*Malus pumila*	Liu 1994
**Fujian**			
Zhangzhou	*T. pakistanensis*	*Litchi chinensis*	Xu and Decraemer 1995
Fuzhou	*T. pakistanensis*	*Dimocarpus longan*	Liu and Zhang 1999
Putian	*T. pakistanensis*	*Dimocarpus longan*	Liu and Zhang 1999
Fuzhou	*T. pakistanensis*	*Canarium album*	Zhang et al. 2002
Xiamen	*T. pakistanensis*	*Ficus carica*	Pan et al. 2000
Nan′an	*N. minor*	*Myrica rubra*	Zhang and Chen 1994
Fuzhou	*T. pakistanensis*	*Dimocarpus longan*	This study
Fuzhou	*T. pakistanensis*	*Eriobotrya japonica*	This study
Fuzhou	*T. pakistanensis*	*Citrus reticulata*	This study
Fuzhou	*T. pakistanensis*	*Ilex chinensis*	This study
Fuzhou	*P. porosus*	*Citrus reticulata*	This study
Fuzhou	*T. pakistanensis*	*Dimocarpus longan*	This study
Xiamen	*T. pakistanensis*	*Xylosma congestum*	This study
Xiamen	*P. porosus*+ *N. minor*	*Dimocarpus longan*	This study
Xiamen	*P. porosus*	*Dimocarpus longan*	This study
Xiamen	*T. pakistanensis*	*Dimocarpus longan*	This study
Zhangzhou	*P. porosus*	*Litchi chinensis*	This study
Zhangzhou	*T. pakistanensis*	*Dimocarpus longan*	This study
Zhangzhou	*T. pakistanensis*	*Dimocarpus longan*	This study
Zhangzhou	*T. pakistanensis*	*Dimocarpus longan*	This study
Zhangzhou	*T. pakistanensis*	*Litchi chinensis*	This study
Zhangzhou	*T. pakistanensis*	*Dimocarpus longan*	This study
**Anhui**			
Huangshan	*P. porosus*	*Boehmeria nivea*	Liu and Cheng 1990
Shexian	*T. pakistanensis*	*Boehmeria nivea*	Xu and Decraemer 1995
**Hunan**			
Changsha	*P. porosus*	*Averrhoa carambola*	Wang et al. 1996
Changsha	*P. porosus*	*Pinus massoniana*	This study

**Table 3.** Measurements of females of *Trichodorus*, *Paratrichodorus* and *Nanidorus* species from China (all measurements in μm).

Species	*T. nanjingensis*	*T. pakistanensis*	*T. cedarus*	*P. porosus*	*N. renifer*	*N. minor*
Location	Beijing	Fuzhou Fujian Province	Hangzhou Zhejiang Province	Xiamen Fujian Province	Jinhua Zhejiang Province	Danzhou Hainan Province
n	10	11	13	25	19	6
L	1099.1 ± 117.7 (836–1284)	1051.7 ± 91.2 (883.5–1175)	749.2 ± 56.5 (680–843)	731.9 ± 113.4 (498.5–898)	538.1 ± 36.8 (466.5–607.5)	645.8 ± 16.9 (632–678)
Body diam	52.9 ± 7.7 (389–63)	49.2 ± 5.9 (39–59)	45.9 ± 4.3 (34–52)	48.0 ± 6.6 (36–58)	24.3 ± 2.9 (20.6–29.3)	31.6 ± 3.6 (27–35)
Pharynx	170.6 ± 16.7 (155–214)	159.9 ± 11.1 (139–172)	176.7 ± 10.8 (161–197)	143.9 ± 19.0 (103–173)	111.4 ± 10.3 (91–133)	129.7 ± 2.6 (126–133)
Onchiostyle	54.3 ± 4.5 (47–63)	50.7 ± 2.1 (47–54)	66.2 ± 2.3 (62–-70)	53.5 ± 1.7 (49–57)	36.3 ± 1.7 (33–41)	35.3 ± 0.7 (34.5–36.5)
Ant. end to S-E pore	139.7 ± 7.9 (137–154)	116.5 ± 11.6 (110–134)	122.8 ± 8.0 (111–-135)	–	105	–
Ant. genital branch	217.2 ± 35.5 (172–274)	185.8 ± 35.5 (141–265)	186.1 ± 34.2 (141–266)	122.6 ± 22.4 (91–153)	114.2 ± 16.7 (69.5–136)	120.2 ± 31.7 (90.5–154)
Post. genital branch	224.6 ± 30.9 (160–269)	172.6 ± 39.8 (132–262)	178.5 ± 26.5 (144–221)	149.0 ± 32.0 (104–212)	105.3 ± 14.3 (67–133)	98.5 ± 25.4 (70–119)
a	21.1 ± 2.9 (15.6–25.7)	21.6 ± 2.8 (18.2–26.6)	16.5 ± 1.8 (14.1–20.3)	15.2 ± 1.5 (12.4–19.2)	22.4 ± 2.1 (17.7–25.5)	20.7 ± 2.4 (17.9–23.6)
b	6.5 ± 0.9 (4.7–7.7)	6.6 ± 0.8 (5.6–8.4)	4.2 ± 0.3 (3.8–4.6)	5.1 ± 0.6 (4.2–6.3)	4.9 ± 0.5 (4.1–6.0)	5.0 ± 0.1 (4.9–5.2)
V	56.1 ± 2.4 (50.3–57.9)	56.6 ± 0.9 (55.1–57.8)	57.7 ± 1.1 (55.8–59.7)	54.8 ± 2.2 (50.9–57.7)	56.4 ± 1.7 (53.7–59.1)	56.3 ± 0.0 (52.5–58.8)
Length of vagina	18.8 ± 1.7 (16–21)	17.1 ± 0.9 (15–18)	18.5 ± 1.6 (17–22)	8.6 ± 1.3 (6–10)	6.1 ± 0.7 (5–7)	11
Size of vaginal pieces	2	2.3 ± 0.2 (2–3)	2	1	1.5 ± 0.1 (1–2)	2

**Table 4.** Measurements of males of *Trichodorus* species from China (all measurements in μm).

Species	*T. nanjingensis*	*T. pakistanensis*	*T. cedarus*
Location	Beijing	Fuzhou Fujian Province	Hangzhou Zhejiang Province
n	16	9	14
L	1011.7 ± 122.9 (731–1163)	933.7 ± 91.4 (812–1074)	765.3 ± 54.3 (650–862)
Body diam	48.7 ± 9.1 (35.5–60)	43.7 ± 6.3 (34–53)	42.8 ± 4.2 (36.9–49.1)
Pharynx	160.6 ± 14.6(132–190)	159.9 ± 11.5 (147–177)	174.5 ± 7.3 (160–187.5)
Onchiostyle	54.2 ± 3.4 (49–59)	50.9 ± 2.5 (48–54)	67.7 ± 1.5 (65–70)
Ant. end to S-E pore	127.6 ± 12.8 (113–142)	124.8 ± 8.5 (113–135)	127.0 ± 7.2 (117–142)
CP1-CP2	12.3 ± 1.4(11–15)	11.8 ± 2.9 (8–17)	10.1 ± 1.7 (7–14)
CP2-CP3		15.1 ± 0.7 (14–16)	8.9 ± 1.5 (5–10)
CP3 to S-E pore (CP2 to S-E pore for *T. nanjingensis*)	14.2 ± 3.4 (7–19)	6.4 ± 1.0 (5–8)	7.6 ± 3.8 (2.5–17)
a	21.1 ± 2.5 (17.0–25.3)	21.7 ± 3.3 (17.0–26.8)	18.0 ± 1.9 (15.1–22.2)
b	6.3 ± 1.0 (4.5–8.7)	5.9 ± 0.5 (4.8–6.4)	4.4 ± 0.3 (4.1–4.9)
T	61.0 ± 4.2 (56.7–68.0)	61.7 ± 3.8 (57.1–69.5)	64.6 ± 3.8 (57.4–69.6)
Spicules	48.3 ± 2.7 (43–53)	54.4 ± 2.8 (48.5–57.2)	44.6 ± 2.5 (40–48)
Gubernaculum	21.3 ± 1.7 (19–24)	15.7 ± 1.6 (12–18)	20.3 ± 1.3 (19–22)
Cloaca to SP1	27.7 ± 3.4 (21.5–34)	37.5 ± 3.6 (33–44)	27.5 ± 2.0 (24–30.5)
SP1-SP2	35.5 ± 4.2 (27–40)	45.2 ± 5.8 (39.5–59)	40.2 ± 5.2 (30.5–51)
SP2-SP3	50.3 ± 10.6 (30.5–65)	51.8 ± 8.3 (36–65)	44.9 ± 4.0 (38–50)

## Discussion

Among trichodorids, *Trichodorus*, *Nanidorus* and *Paratrichodorus* are cosmopolitan genera, species of those genera have been reported from all the continents except Antarctica (Decraemer and Robbins 2007). Regional endemicity has been observed for *Trichodorus* and *Paratrichodorus* species. Subbotin et al. (2019) stated that Californian populations of *Trichodorus* may be endemic originating in the same region, and hypothesized that this is an apparent centre of speciation, in addition to the Iberian Peninsula (Decraemer et al. 2013) and Irano-Anatolian region (Pedram et al. 2015, 2017, Asghari et al. 2018). The distribution of trichodorids throughout Asia (except for Iran) is not well documented but in the distribution data of EPPO 2014, trichodorids are either present or widespread in Afghanistan, Bahrain, China, India, Indonesia, Japan, Korea, Turkey, and Uzbekistan. Taxonomic and faunistic records presently list 10 trichodorids (six *Trichodorus* spp., two *Paratrichodorus* spp. and two *Nanidorus* spp.) from China, which represents a comparatively low diversity, and possibly reflects the relatively few studies conducted; so far about half of the territory of the country has been observed for this nematode group with different intensity of sampling. *Trichodorus hangzhouensis* sp. nov. seems to represent another endemic for China in addition to *T. nanjingensis* and *T. guangzhouensis* which are reported only for this country so far.

**Figure 7.**
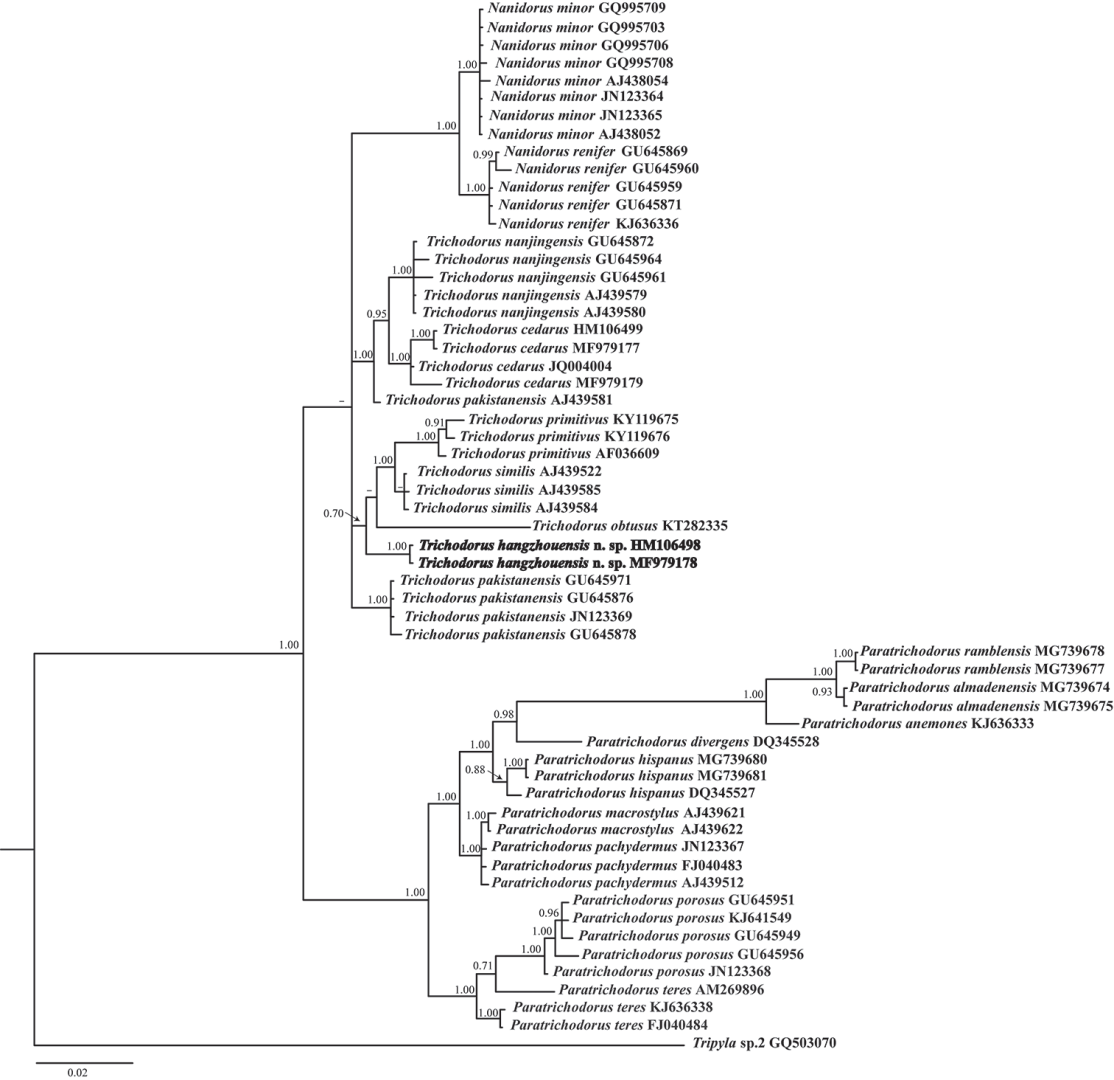
Phylogenetic relationships of *Trichodorus hangzhouensis* sp. nov. and other trichodorid species based on partial 18S rRNA gene sequences. The Bayesian tree was inferred under the general time-reversible model of sequence evolution with correction for invariable sites and a gamma-shaped distribution (GTR+I+G). *Tripyla* sp. served as an outgroup species. Posterior probability values exceeding 70% are given on appropriate clades.

The D2-D3 region of the 28S rDNA gene has been shown to be of importance in trichodorid molecular taxonomy (Subbotin et al. 2019). The phylogenetic analysis inferred from this gene sequences revealed four highly supported clades. The first is the largest one and consists of *Trichodorus* species from Europe, Asia and the USA including *T. hangzhouensis* sp. nov., and corresponds to the Clade I according to Subbotin et al. (2019), the second clade includes *Nanidorus* species and *Trichodorus* species from Asia (corresponds to the Clade II (Subbotin et al. 2019)), the third clade consists of *Paratrichodorus* species distributed in USA, Europe and Asia (Clade III in Subbotin et al. 2019), the fourth clade includes *Trichodorus* and *Monotrichodorus* from the USA (Clade IV and V according to the same authors). These results are consistent also with other previously published studies (Asghari et al. 2018, Decraemer et al. 2019). It is interesting to mention that the three species occurring in southeastern Asia (*T. cedarus*, *T. nanjingensis* and *T. japonicus*) form a highly supported subclade within Clade II, while the new species is part of another phylogenetically more distant group (Clade I). However, in the ITS2 tree the position of *T. nanjingensis* differs substantially.

**Figure 8.**
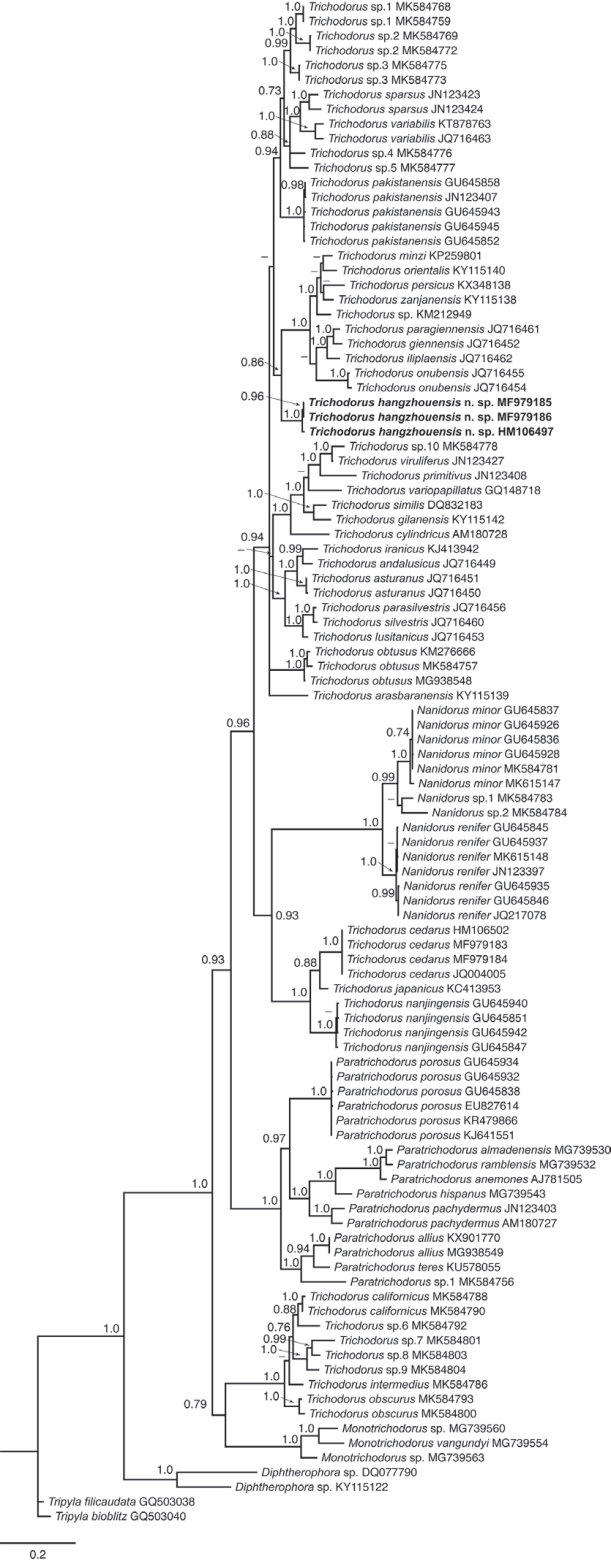
Phylogenetic relationships of *Trichodorus hangzhouensis* sp. nov. and other trichodorid species based on D2-D3 28S rRNA gene sequences. The Bayesian tree was inferred under the general time-reversible model of sequence evolution with correction for invariable sites and a gamma-shaped distribution (GTR+I+G). *Tripylina tamaki* served as an outgroup species. Posterior probability values exceeding 70% are given on appropriate clades.

In addition, the trichodorid species molecularly characterized during this study (*T. nanjingensis*, *T. cedarus*, *T. pakistanensis*, *P. porosus*, *N. minor*, *N. renifer*) clustered with the known species from different countries; these results further validated their identity. It is also noted that the position of *T. hangzhouensis* sp. nov. differs more or less in the phylogenetic trees based on the different gene sequences, and this could be also caused by the incomplete sequence data for a given species.

All present, and most previous, phylogenetic reconstructions inferred from three different gene sequences (18S, D2D3 28S and ITS2) showed that *Nanidorus* and *Paratrichodorus* species each formed highly supported clades. *Trichodorus* species studied molecularly so far take three different positions based on D2D3 28S r RNA gene sequences: i) the large part containing only *Trichodorus* species and forming Clade I sensu Subbotin et al. (2019); ii) three species of southeastern Asian origin clustering together with *Nanidorus* species and iii) Californian species forming a highly supported clade.

**Figure 9.**
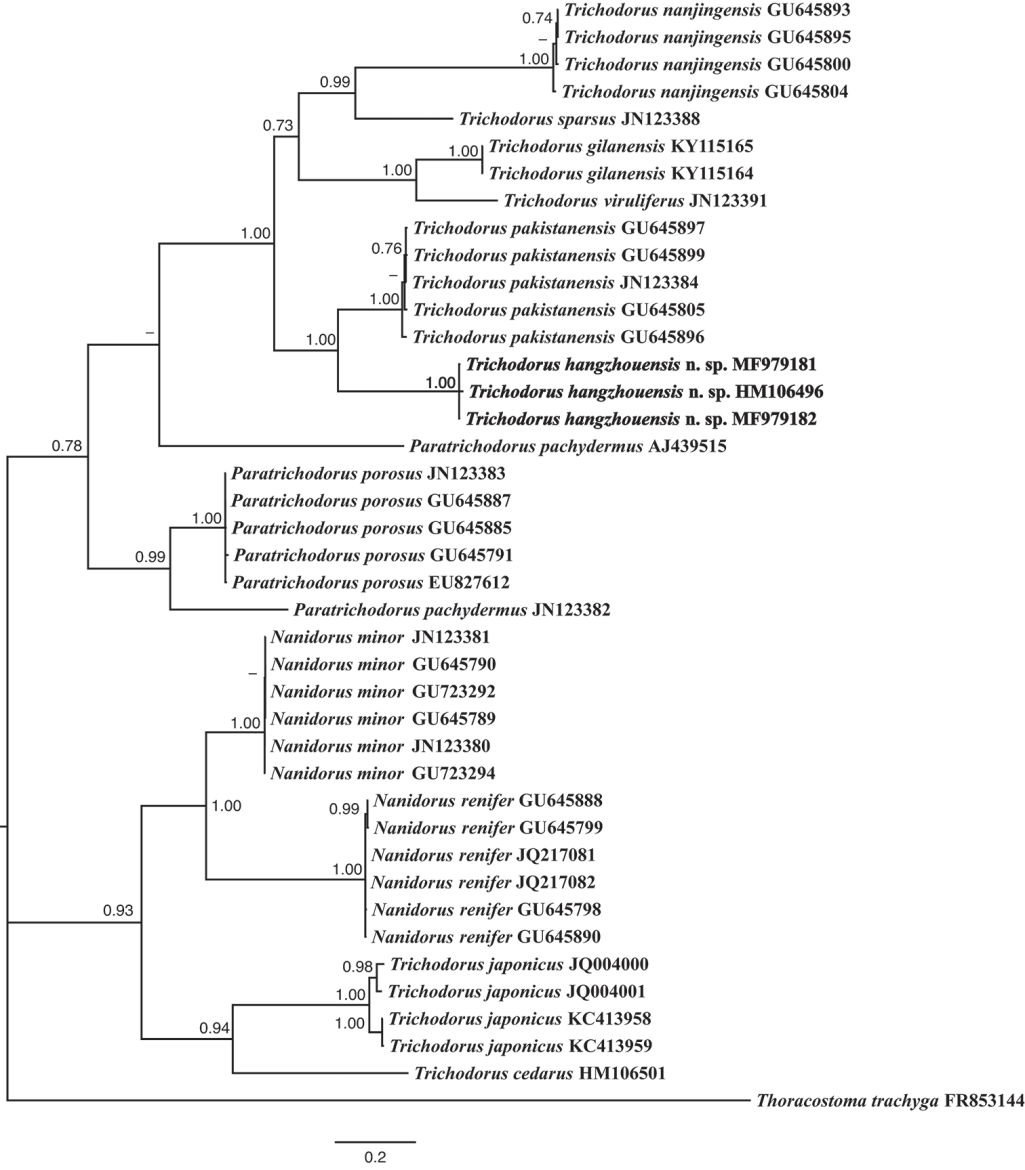
Phylogenetic relationships of *Trichodorus hangzhouensis* sp. nov. and other trichodorid nematodes based on ITS2 sequences. The Bayesian tree was inferred under the general time-reversible model of sequence evolution with correction for invariable sites and a gamma-shaped distribution (GTR+I+G). *Thoracostoma trachyga* served as an outgroup species. Posterior probability values exceeding 70% are given on appropriate clades.

All the three aforementioned genera occur in China. From our observations, trichodorids seem not host specific and can be found in various types of ecosystems. The occurrence of *Trichodorus*, *Nanidorus* and *Paratrichodorus* recovered from soils in China is quite low (4.1%) compared to trichodorid occurrence in other countries such as Great Britain (22%), Italy (9.6%), Iran (7%), Belgium (19.6), Portugal (32.6%) and Slovak Republic (33%) (Alphey and Boag 1976, Roca and Lamberti 1985, De Waele and Sturhan 1987, De Waele and Coomans 1991, Almeida 1993, Lišková and Sturhan 1999).

In the past surveys concerning the stubby root nematodes, this group is reported being generally in somewhat low densities (Aballay and Eriksson 2006). The low density of trichodorids populations in the soil could be related to the sampling strategies (depth and intensity of sampling) or studied plants (crops or natural vegetation). Boag (1981) suggested that the distribution of trichodorids is correlated with soil moisture, particle size structure and seasonal fluctuation of temperature. De Waele and Coomans (1991) also recognized that geographic distribution of certain trichodorid species may be influenced by their habitat and found a relatively high presence of populations in soil with a pH <5.5.

In conclusion, this study provides a morphological and molecular characterization of *T. hangzhouensis* sp. nov. and three known trichodorid species together with updated records of this group in China. Among 164 populations recovered in China, the highest number of records is for *P. porosus* (42.6%) followed by *T. nanjingensis* (39.6%). The systematics and diagnostics of trichodorid nematodes are important because of regulatory and management issues attributed to this group of nematodes being vectors of tobra viruses. Thus, updated descriptions based on sufficient examination material and accurately identified specimens, coupled with molecular analysis are necessary for better understanding of the current distribution and host association of this complex group of nematodes.

## Supplementary Material

XML Treatment for Trichodorus hangzhouensis

## Supplementary materials

Supplementary material 1 Table S1. Sequences of nematode species used for the phylogenetic analyses Data type species data https://binary.pensoft.net/file/428640This dataset is made available under the Open Database License (http://opendatacommons.org/licenses/odbl/1.0/). The Open Database License (ODbL) is a license agreement intended to allow users to freely share, modify, and use this Dataset while maintaining this same freedom for others, provided that the original source and author(s) are credited.Xuqing Li, Munawar Maria, Ruihang Cai, Eda Marie Barsalote, Vlada Peneva, Jingwu Zheng
