# Supplementary material for: Distribution of trichodorid species in mainland China with description of Trichodorus hangzhouensis sp. nov. (Nematoda, Triplonchida)
Source: Zookeys. 2020 Jul 3;945:163–89. doi: 10.3897/zookeys.945.50424 (PMC7351824; doi:10.3897/zookeys.945.50424)
Supplement: Supplementary material 1 — Table S1. Sequences of nematode species used for the phylogenetic analyses [file zookeys-945-163-s001.doc]

**Supplementary Table S1. Sequences of nematode species used for the phylogenetic analyses.**

| **Species** | **18S** | **28S** | **ITS** |  | **Location** |
| --- | --- | --- | --- | --- | --- |
| *T. nanjingensis* | GU645961 | GU645851 | GU645804 | This study | Zhejiang, China |
|  | — | GU645942 | GU645895 | This study | Zhejiang, China |
|  | GU645872 | GU645847 | GU645800 | This study | Beijing, China |
|  | GU645964 | GU645940 | GU645893 | This study | Beijing, China |
|  | AJ439580 | — | — | GenBank | Beijing, China |
|  | AJ439579 | — | — | GenBank | Beijing, China |
| *T. pakistanensis* | GU645971 | GU645858 | GU645896 | This study | Chongqing, China |
|  | GU645876 | GU645943 | GU645897 | This study | Fujian, China |
|  | GU645878 | GU645945 | GU645899 | This study | Fujian, China |
|  | — | GU645852 | GU645805 | This study | Yunnan,China |
|  | JN123369 | JN123407 | JN123384 | GenBank | Rajot, India |
|  | AJ439581 | — | — | GenBank | Zhejiang, China |
| *T. cedarus* | HM106499 | HM106502 | HM106501 | This study | Zhejiang, China |
|  | MF979177 | MF979183 | — | This study | Zhejiang, China |
|  | MF979179 | MF979184 | — | This study | Zhejiang, China |
|  | JQ004004 | JQ004005 | — | GenBank | Japan |
| ***T. hangzhouensis* sp. n.** | **HM106498** | **HM106497** | **HM106496** | **This study** | **Zhejiang, China** |
|  | **MF979178** | **MF979186** | **MF979182** | **This study** | **Zhejiang, China** |
|  | — | **MF979185** | **MF979181** | **This study** | **Zhejiang, China** |
| *P. porosus* | GU645949 | EU827614 | EU827612 | This study | Zhejiang, China |
|  | GU645951 | GU645838 | GU645791 | This study | Yunnan, China |
|  | GU645956 | GU645934 | GU645887 | This study | Fujian, China |
|  | — | GU645932 | GU645885 | This study | Guangdong, China |
|  | — | KR479866 | — | GenBank | Tehran, Iran |
|  | KJ641549 | KJ641551 | — | GenBank | Shenzhen, China |
|  | JN123368 | — | JN123383 | GenBank | California, USA |
| *N. renifer* | GU645959 | GU645845 | GU645798 | This study | Zhejiang, China |
|  | GU645960 | GU645846 | GU645799 | This study | Zhejiang, China |
|  | GU645871 | GU645937 | GU645890 | This study | Zhejiang, China |
|  | GU645869 | GU645935 | GU645888 | This study | Zhejiang, China |
|  | KJ636336 | — | — | GenBank | Gelderland, Netherlands |
|  | — | JQ217078 | JQ217082 | GenBank | China |
|  | — | — | JQ217081 | GenBank | China |
|  | — | MK615148 | — | GenBank | Florida, USA |
|  | — | JN123397 | — | GenBank | Florida, USA |
| *N. minor* | GQ995703 | GU645926 | GU723292 | This study | Hainan, China |
|  | GQ995706 | GU645836 | GU645789 | This study | Yunnan, China |
|  | GQ995708 | GU645928 | GU723294 | This study | Yunnan, China |
|  | GQ995709 | GU645837 | GU645790 | This study | Fujian, China |
|  | AJ438052 | — | — | GenBank | Portugal |
|  | AJ438054 | — | — | GenBank | Brazil |
|  | JN123364 | — | JN123380 | GenBank | California, USA |
|  | JN123365 | — | JN123381 | GenBank | California, USA |
|  | — | MK584781 | — | GenBank | California, USA |
|  | — | MK615147 | — | GenBank | Florida, USA |
| *T. andalusicus* | — | JQ716449 | — | GenBank | Córdoba, Spain |
| *T. arasbaranensis* | — | KY115139 | — | GenBank | Iran |
| *T. asturanus* | — | JQ716451 | — | GenBank | Oviedo, Spain |
|  | — | JQ716450 | — | GenBank | Oviedo, Spain |
| *T. californicus* | — | MK584788 | — | GenBank | California, USA |
|  | — | MK584790 | — | GenBank | California, USA |
| *T. cylindricus* | — | AM180728 | — | GenBank | UK |
| *T. giennensis* | — | JQ716452 | — | GenBank | Jaén, Spain |
| *T. gilanensis* | — | KY115142 | — | GenBank | Iran |
|  | — | — | KY115165 | GenBank | Iran |
|  | — | — | KY115164 | GenBank | Iran |
| *T. iliplaensis* | — | JQ716462 | — | GenBank | Huelva, Spain |
| *T. intermedius* | — | MK584786 | — | GenBank | California, USA |
| *T. iranicus* | — | KJ413942 | — | GenBank | Iran |
| *T. japonicus* | — | KC413953 | — | GenBank | Japan |
|  | — | — | JQ004000 | GenBank | Japan |
|  | — | — | JQ004001 | GenBank | Japan |
|  | — | — | KC413958 | GenBank | Japan |
|  | — | — | KC413959 | GenBank | Japan |
| *T. lusitanicus* | — | JQ716453 | — | GenBank | Portugal |
| 1. *minzi* | — | KP259801 | — | GenBank | Iran |
| *T. obscurus* | — | MK584793 | — | GenBank | California, USA |
|  | — | MK584800 | — | GenBank | California, USA |
| *T. obtusus* | KT282335 | — | — | GenBank | Carolina, USA |
|  | — | KM276666 | — | GenBank | Wilmington, USA |
|  | — | KM584757 | — | GenBank | USA |
|  | — | MG938548 | — | GenBank | USA |
| *T. onubensis* | — | JQ716455 | — | GenBank | Huelva, Spain |
|  | — | JQ716454 | — | GenBank | Huelva, Spain |
| *T. orientails* | — | KY115140 | — | GenBank | Iran |
| *T. paragiennensis* | — | JQ716461 | — | GenBank | Huelva, Spain |
| *T. parasilvestris* | — | JQ716456 | — | GenBank | Huelva, Spain |
| *T. persicus* | — | KX348138 | — | GenBank | Iran |
| *T. primitivus* | KY119675 | — | — | GenBank | Ireland |
|  | KY119676 | — | — | GenBank | Ireland |
|  | AF036609 | — | — | GenBank | Uknown |
|  | — | JN123408 | — | GenBank | Ratiskovice, Czech Republic |
| *T. silvestris* | — | JQ716460 | — | GenBank | Huelva, Spain |
| *T. similis* | AJ439522 | — | — | GenBank | Kinshaldy, UK |
|  | AJ439584 | — | — | GenBank | Plovdiv, Bulgaria |
|  | AJ439585 | — | — | GenBank | Mesta Bacoruda, Bulgaria |
|  | — | DQ832183 | — | GenBank | Czech Republic |
| *T. sparsus* | — | JN123423 | — | GenBank | Vojtechov, Czech Republic |
|  | — | JN123424 | JN123388 | GenBank | Borek, Czech Republic |
| *T. variabilis* | — | KT878763 | — | GenBank | Tehran, Iran |
|  | — | JQ716463 | — | GenBank | Epirus, Greece |
| *T. variopapillatus* | — | GQ148718 | — | GenBank | Krivoklat, Czech Republic |
| *T. viruliferuss* | — | JN123427 | — | GenBank | Ratiskovice, Czech Republic |
|  | — | — | JN123391 | GenBank | Vysoka, Czech Republic |
| *T. zanjanensis* | — | KY115138 | — | GenBank | Iran |
| *Trichodorus* sp. | — | KM212949 | — | GenBank | unknown, possibly Asia |
| *Trichodorus* sp.1 | — | MK584768 | — | GenBank | California, USA |
| *Trichodorus* sp.1 | — | MK584759 | — | GenBank | California, USA |
| *Trichodorus* sp.2 | — | MK584769 | — | GenBank | California, USA |
| *Trichodorus* sp.2 | — | MK584772 | — | GenBank | California, USA |
| *Trichodorus* sp.3 | — | MK584775 | — | GenBank | California, USA |
| *Trichodorus* sp.3 | — | MK584773 | — | GenBank | California, USA |
| *Trichodorus* sp.4 | — | MK584776 | — | GenBank | California, USA |
| *Trichodorus* sp.5 | — | MK584777 | — | GenBank | Nevada, USA |
| *Trichodorus* sp.6 | — | MK584792 | — | GenBank | California, USA |
| *Trichodorus* sp.7 | — | MK584801 | — | GenBank | California, USA |
| *Trichodorus* sp.8 | — | MK584803 | — | GenBank | California, USA |
| *Trichodorus* sp.9 | — | MK584804 | — | GenBank | California, USA |
| *Trichodorus* sp.10 | — | MK584778 | — | GenBank | Mexico |
| *P. allius* | — | KX901770 | — | GenBank | ND, USA |
|  | — | MG938549 | — | GenBank | USA |
| *P. almadenensis* | MG739674 | — | — | GenBank | Sevilla, Spain |
|  | MG739675 | — | — | GenBank | Huelva, Spain |
|  |  | MG739530 | — | GenBank | Niebla, Spain |
| *P. anemones* | KJ636333 | — | — | GenBank | Gelderland, Netherlands |
|  | — | AJ781505 | — | GenBank | Yorkshire, UK |
| *P. divergens* | DQ345528 | — | — | GenBank | Alijo, Portugal |
| *P. hispanus* | DQ345527 | — | — | GenBank | Braga, Portugal |
|  | MG739680 | — | — | GenBank | Huelva, Spain |
|  | MG739681 | MG739543 | — | GenBank | Huelva, Spain |
| *P. macrostylus* | AJ439622 | — | — | GenBank | Svatusa, Slovakia |
|  | AJ439621 | — | — | GenBank | Svatusa, Slovakia |
| *P. pachydermus* | JN123367 | — | — | GenBank | Jesen, Czech Republic |
|  | FJ040483 | — | — | GenBank | Unknown |
|  | — | JN123403 | — | GenBank | Obory, Czech Republic |
|  | — | AM180727 | — | GenBank | UK |
|  | AJ439512 | — | — | GenBank | UK |
|  | — | — | AJ439515 | GenBank | Woodhill, United Kingdom |
|  | — | — | JN123382 | GenBank | Jesen, Czech Republic |
| *P. ramblensis* | MG739678 | — | — | GenBank | Huelva, Spain |
|  | MG739677 | — | — | GenBank | Málaga, Spain |
|  | — | MG739532 | — | GenBank | Córdoba, Spain |
| *P. teres* | AM269896 | — | — | GenBank | Greece |
|  | KJ636338 | — | — | GenBank | Netherlands |
|  | FJ040484 | — | — | GenBank | Netherlands |
|  | — | KU578055 | — | GenBank | Poland |
| *Paratrichodorus* sp.1 | — | MK584756 | — | GenBank | Anapa, Russia |
| *Nanidorus* sp.1 | — | MK584783 | — | GenBank | California, USA |
| *Nanidorus* sp.2 | — | MK584784 | — | GenBank | Malaysia |
| *Monotrichodorus* sp. | — | MG739560 | — | GenBank | Ecuador |
|  | — | MG739563 | — | GenBank | Ecuador |
| *Monotrichodorus vangundyi* | — | MG739554 | — | GenBank | Costa Rica |
| *Diphtherophora* sp. | — | DQ077790 | — | GenBank | California, USA |
|  | — | KY115122 | — | GenBank | Iran |
| *Tripyla bioblitz* | — | GQ503040 | — | GenBank | New Zealand |
| *Tripyla filicaudata* | — | GQ503038 | — | GenBank | New Zealand |
| *Tripyla* sp.2 | GQ503070 | — |  | GenBank | Auckland, New Zealand |
| *Thoracostoma trachyga* | — | — | FR853144 | GenBank | California, USA |
